# Development and Validation of a Real-Time PCR Assay for Rapid Detection of Two-Spotted Spider Mite, *Tetranychus urticae* (Acari: Tetranychidae)

**DOI:** 10.1371/journal.pone.0131887

**Published:** 2015-07-06

**Authors:** Dongmei Li, Qing-Hai Fan, David W. Waite, Disna Gunawardana, Sherly George, Lalith Kumarasinghe

**Affiliations:** Plant Health and Environment Laboratory, Ministry for Primary Industries, Auckland, New Zealand; Nanjing Agricultural University, CHINA

## Abstract

Spider mites of the genus *Tetranychus* are difficult to identify due to their limited diagnostic characters. Many of them are morphologically similar and males are needed for species-level identification. *Tetranychus urticae* is a common interception and non-regulated pest at New Zealand’s borders, however, most of the intercepted specimens are females and the identification was left at *Tetranychus* sp. Consequently, the shipments need to be fumigated. DNA sequencing and PCR-restriction fragment length polymorphism (PCR-RFLP) protocols could be used to facilitate the accurate identification. However, in the context of border security practiced in New Zealand, insect identifications are required to be provided within four hours of receiving the samples; thus, those molecular methods are not sufficient to meet this requirement. Therefore, a real-time PCR TaqMan assay was developed for identification of *T*. *urticae* by amplification of a 142 bp Internal Transcribed Spacer (ITS) 1 sequence. The developed assay is rapid, detects all life stages of *T*. *urticae* within three hours, and does not react with closely related species. Plasmid DNA containing ITS1 sequence of *T*. *uritcae* was serially diluted and used as standards in the real-time PCR assay. The quantification cycle (*Cq*) value of the assay depicted a strong linear relationship with *T*. *urticae* DNA content, with a regression coefficient of 0.99 and efficiency of 98%. The detection limit was estimated to be ten copies of the *T*. *urticae* target region. The assay was validated against a range of *T*. *urticae* specimens from various countries and hosts in a blind panel test. Therefore the application of the assay at New Zealand will reduce the unnecessary fumigation and be beneficial to both the importers and exporters. It is expected that the implementation of this real-time PCR assay would have wide applications in diagnostic and research agencies worldwide.

## Introduction

The family Tetranychidae is one of the most important families of the Acari in terms of economic impact. It comprises over 1,200 described species in about 70 genera [1]. The genus *Tetranychus* includes 149 species, some of which are of high economic and quarantine importance in agriculture [1]. They occur throughout the world and are found on virtually all major food crops and ornamental plants, often causing serious injury to, or death of, the host [2]. *Tetranychus urticae* Koch, 1836, two spotted spider mite, is a highly polyphagous species which can feed on more than 1,100 plant species, from more than 140 different plant families [3]. It is a major pest in greenhouse and field crops, including peppers, tomatoes, potatoes, beans, maize and strawberries and ornamental plants such as roses.

Morphological identification of tetranychid species is difficult because the number of potential diagnostic characters is limited, partly due to their small size (~0.4mm long), and key traits often exhibit large phenotypic plasticity [4–7]. As a result, many species cannot be distinguished on the basis of external morphology [8]. Species level identification of *Tetranychus* can only be performed by microscopic examination of the shape of the male genitalia [9]. However, most specimens intercepted at the New Zealand border are females, nymphs or eggs. Males are less abundant due to the female-biased sex ratio of spider mites [10,11]. Under this circumstance, over 50% of the intercepted *Tetranychus* were not able to be identified to species level using morphological characters at New Zealand’s borders. However, some *Tetranychus* species are quarantine pests, such as *T*. *evansi*, while others are not, such as *T*. *urticae*, therefore, accurate identification of *Tetranychus* species is essential for the implementation of phytosanitary measurement in New Zealand border.

Molecular methods have revolutionised insect taxonomy and systematics [12–14], and are increasingly being applied to ticks and mites [5,15–20]. DNA sequences of ITS and COI have been widely used as barcodes to distinguish *Tetranychus* species [6,16,17,21,22]. Recently, six *Tetranychus* mitochondrial genomes [23] have been sequenced, and the genome comparison indicated that the red and green forms of *T*. *urticae* are synonymous species [23]. The molecular diagnostic method, PCR-restriction fragment length polymorphism (PCR-RFLP) targeting the ITS region has been applied in identification of *Tetranychus* species, which could be used to distinguish up to 14 species [24–26]. The PCR-RFLP method is currently in use in the Japanese import plant quarantine division [24], which takes at least 8 hours to obtain results, as it requires more handling steps than conventional PCR and real-time PCR. Real-time PCR uses fluorescent marker molecules to track the accumulation of PCR product during the reaction and thus there is no need for additional analysis post-PCR reaction. Furthermore, real-time PCR assay combines the annealing and extension steps of the reaction, resulting in a shorter reaction time than conventional PCR. In the context of border security in New Zealand, entomological identifications are required within four hours of receiving the samples and the existing restriction enzyme-based protocols are not sufficient to meet this requirement. Therefore, developing a real-time PCR assay for *Tetranychus* species is needed in order to provide rapid identification for the intercepted material while avoiding the time issue associated with current available methods.

This study reports the development of a rapid real-time TaqMan assay to distinguish *T*. *urticae* from other closely related species. Here we report the assay development and performance indicators such as analytical specificity and sensitivity, diagnostic specificity, repeatability and reproducibility. The assay was further validated using a blind panel test to simulate the New Zealand quarantine framework.

## Materials and Methods

### 1.1 Sample collection and identification

Mites from laboratory reared colonies, field collections and border interceptions were used in this study (Tables 1 and 2). Specimens from overseas laboratory colonies preserved in ethanol (70–96%) were obtained from Japan, China and Australia. The intercepted material collected in Plant Health and Environment Laboratory (PHEL) in New Zealand used in this study were stored at -20°C freezer. Identifications of all the specimens were reconfirmed morphologically and/or molecularly.

**Table 1:** Sampling details for target organism, *T*. *urticae* used in specificity tests of the real-time PCR assay.

Sample ID	Origin	Host	DNA extraction^a^	Mean *Cq* ^b^
MQ24MQ25	Japan	Unknown	QG	19.2720.88
Q9Q10Q12	India	*Rosa* spp.	QG	19.3422.7126.82
MQ36	Netherland	*Rosa* spp.	QG	23.82
MQ40MQ41	India	*Rosa* spp.	QG	31.1125.70
MQ42*MQ43*MQ52*	Unknown	Unknown	QG	22.3318.9925.35
MQ44*	NZ	*Erythrina* sp.	QG	24.78
MQ51MK5	USA	*Fragaria* spp.	QGKF	23.7727.64
MQ53	Australia	*Citrus unshiu*	QG	22.2631.8429
MK3MK4			KFKF	.53
MQ54MQ55	Australia	*Phaseolus* sp.	QG	19.8116.91
MQ61*MQ62*	Colombia	*Rosa* spp.	QG	29.6629.39
MQ63*	Malaysia	*Gypsophila paniculata*	QG	18.86
MQ64	Colombia	*Rosa* spp.	QG	28.08
MQ65	Colombia	*Rosa* spp.	QG	34.76
MQ66*	Colombia	*Rosa* spp.	QG	21.29
MZ5MZ6	India	*Rosa* spp.	ZR	20.1720.40
MP3MP4	India	*Rosa* spp.	PG	26.1624.97
MQa*	New Zealand	*Malus* spp.	QG	21.06
MQb*	USA	*Pyrus* spp.	QG	22.98
MQc*	USA	*Pyrus* spp.	QG	27.32
MQ34MQ35	Japan	Unknown	QG	20.44

All the samples were identified with morphological first and then confirmed by DNA sequences. Asterisk (*) denotes samples which could not be identified morphologically and were identified using COI and/or ITS sequences^,^

^**a**^ DNA extractions were performed using several methods, QG = Qiagen DNeasy Blood and Tissue kit; ZR = ZR Tissue & Insect DNA kit (Zymo Research, CA, USA); PG = PrepGEM (ZyGem, USA), KF = Kingfisher Cell and Tissue kit (Thermo Scientific, USA).

^**b**^ All samples were tested in duplicate and all *Cq* values provided are means of the duplicate wells.

**Table 2:** Sampling details for non-target organisms used in specificity tests of the real-time PCR assay. All the samples were tested negative to *T*. *urticae* in the real-time PCR assay in duplicate wells.

Sample ID	Organism^a^	Origin	Host	DNA extraction^b^
MQ22, 23	*T*. *parakanzawai*	Japan	*Pueraria montana*	QG
MQ28, 29	*T*. *kanzawai*	Japan	*Camellia sinensis*	QG
MQ26, 27	*T*. *truncatus*	Japan	Unknown	QG
MQ32, 33	*T*. *ludeni*	Japan	*Solidago virgaurea*	QG
MQ38, 39	*T*. ludeni*	NZ (Auckland)	*Araujia* sp.	QG
MQ47, 49,50	*T*. ludeni*	NZ	*Erythrina* sp.	QG
MZ3, MQA6	*T*. *ludeni*	Australia (Sydney)	*Dahilia* sp.	ZR
MQ30, 31	*T*. *pueraricola*	Japan	*Pueraria montana*	QG
MQ58, 68, MZ1, MQA2	*T*. *evansi*	Australia (Sydney)	*Solamum aviculare*	QG(2), ZR(1), QA(1)
MQ59, 60 MQA1	*T*. *lambi*	Australia (Sydney)	*Cucurbita* sp.	QG(2), QA(1)
MP1, 2	*T*. neocalddonicus*	Fiji	*Moringa oleifera*	PG
MQd	*Etotetranychus sexmaculatus*	NZ (Auckland)	*Aristotelia serrata*	QG

Note:

^**a**^ All the samples were identified with morphological first and then confirmed by DNA sequences. Asterisk (*) denotes samples which could not be identified morphologically and were identified using COI and/or ITS sequences^,^

^**b**^ DNA extractions were performed using several methods, QG = Qiagen DNeasy Blood and Tissue kit; QA = QIAamp DNA micro kit (Qiagen, CA, USA); ZR = ZR Tissue & Insect DNA kit (Zymo Research, CA, USA); PG = PrepGEM (ZyGem, USA).

No specific permits were required for sample collections. No samples were collected in national parks and collection permits were not required. No endangered or threatened species were included in this study. All mites imported to New Zealand were in accordance to the Import Health Standard, Section 22 of the Biosecurity Act 1993, New Zealand Legislation. Ethics approval was not required as insects are not classified as animals for the purposes of the Animal Welfare Act, 1999, New Zealand Legislation.

### 1.2 DNA extraction, PCR and sequencing

Total DNA was extracted using the DNeasy for Blood and Tissue kit (Qiagen, Valencia, CA, USA) as per the manufacturer’s instructions. A single mite was used for each extraction and physical disruption was performed by sterile needles or micro-pestles, and DNA was eluted in 100 μL of AE buffer (Qiagen, Valencia, CA, USA). For some samples as specified in Tables 1 and 2, QIAamp micro kit (Qiagen, Valencia, CA, USA), ZR Tissue & Insect DNA kit (Zymo Research), Kingfisher for cell and tissue kit (Thermo Scientific, USA) and enzymatic prepGem DNA kit (ZyGem Corporation Ltd., Hamilton, New Zealand) were used, as per manufacturer’s instructions.

The primers used for PCR amplification and sequencing of the ITS, COI and D2/D3 regions of the 28S ribosomal RNA (rRNA) gene are listed in S1 Table. Briefly, Primer pairs 18SF1 and HC2R [4,27,28], and Mite B and Primer C [29] were used for the amplification of ~1000 bp ITS1-5.8S-ITS2 and ~600 bp ITS1 regions, respectively. CI J-1718F, COIVERA [17,30,31] and D2A, D3B [32] were also used for the amplification of ~800 bp of COI and 28S rRNA genes, respectively. For all the PCR reactions, each 20 μL reaction consisted of 1× GoTaq master mix (Promega, Madison, WI), 250 nM of each primer, 0.04 μg/μL Bovine Serum Albumin (BSA, Sigma-Aldrich Co.), 2 μL of DNA extract. Cycling conditions were: initial denaturation at 94°C for 2 min, 40 cycles of 94°C for 15 sec, 52°C for 30 sec and 72°C for 45 sec, followed by final extension step of 7 min at 72°C. The amplicons were electrophoresed on 1.2% agarose (in 1x TAE buffer) gel stained with SYBR safe (Life Technologies), and visualised using a Gel Doc Software system (BioRad, Hercules, CA, USA). Amplified products were sequenced bi-directionally using the amplification primers by EcoGene (Auckland, New Zealand) or Macrogen (Seoul, South Korea). The obtained DNA sequences were edited and aligned using Geneious Pro 7.1.5 (Biomatters, Auckland, New Zealand) and BLAST searched against the GenBank database [33] or BOLD database [34] to confirm morphological identifications. The sequences have been submitted to GenBank under accession numbers KP744522- KP744535 (S2 Table).

### 1.3 Assay design

DNA sequences of ITS, COI and D2/D3 region from *T*. *urticae* and closely related species were aligned using the in-built aligner in Geneious Pro 7.1.5. Regions that showed differences specific to *T*. *uritcae* were selected manually and used as the region of interest for the location of specific primers and probes. The primers and probe were manually designed and their secondary structures and thermodynamic properties were checked using Geneious and OligoAnalyzer 3.1 [35]. The designed primers and probes were checked against the alignment in Geneious and BLAST searched in the GenBank database to ensure they would not amplify off-target species.

### 1.4 Real-time PCR optimisation

All real-time PCR reactions were set up on a CFX96 Touch Real-time platform (BioRad). Gradients of temperatures (55–66°C), primer concentrations (300 and 400 nM), probe gradients (125–250 nM), and additional MgCl_2_ (3–5 mM) were used to optimise the PCR conditions. To select an appropriate real-time mastermix for the assay, preliminary comparisons were trialled with commercially available mastermixes: PerfeCTa qPCR ToughMix (Quanta Bioscience), SsoFast Probes Supermix (BioRad) and Platinum Quantitative PCR Supermix-UDG (Life Technologies). The assay was also tested in duplex format with 18S ribosomal RNA (rRNA) gene internal control real-time PCR (Applied Biosystems, CA, USA).

### 1.5 Analytical and diagnostic specificity

The target and non-target specimens and their detailed information are listed in Tables 1 and 2. The analytical specificity is the percentage of samples of known identity of the target species that return a positive outcome in the assay. The diagnostic specificity is the percentage of non-target samples of known identity that return a negative outcome in the assay. All available samples were used to calculate analytical and diagnostic specificity for the assay.

### 1.6 Analytical sensitivity, repeatability and reproducibility

To evaluate the analytical sensitivity of the developed assay, a 592 bp template ITS1 gene region, amplified as described in section 2.2 was used to prepare plasmid standards of known copy number. The amplicon was cloned using the TOPOTA vector cloning kit (Invitrogen, Carlsbad, CA, USA) as per the manufacturer’s instructions. Cloning was performed for two biological samples for *T*. *uritcae*, and two clones containing the correct insert were selected for preparing standards. Plasmid DNA was extracted using the Wizard Plus SV Miniprep (Promega, Madison, WI, USA) and digested with restriction enzyme *Pst* I (BioLab Inc, Lawrenceville, GA) overnight to linearise. The linearised plasmid DNA was quantified using a μDrop plate in MultiSkan GO DNA quantification system (Thermo Scientific, USA) and normalised to a concentration of 10^9^ copies / μL. A dilution series of the plasmid from 10^7^–10 copies was created using sterile water. Analytical sensitivity of the assay was determined using the dilution series with each concentration in triplicate per reaction. Linear regression was performed between the detection threshold (*Cq*) and the log_10_ of the copy number, measuring the fit as *r* ^2^. Amplification efficiencies for individual reactions were calculated using the formula, E = 10^|1/slope|^ and converted to E_%_ by (E-1) × 100 in the R environment version 3.1.1 [36].

Repeatability (intra-run variation) and reproducibility (inter-run variation) for the assay were reported by percent coefficient of variance (%CV) within and between assays. Five samples were tested in duplicate at different times and the %CV for individual sample per run was calculated for estimating repeatability. This data from the two runs were used to calculate %CV between runs as a measure of reproducibility.

### 1.7 Blind panel testing

A total of 25 specimens were provided to the operators with no knowledge of the sample origin and identity. These samples constituted a range of species, various life stages and hosts. The samples were tested against the real-time assay in duplicate, positive and no template controls were included. These samples were tested with *T*. *urticae*-specific real-time assay under the optimal condition, and also with a TaqMan 18S internal control real-time PCR (Applied Biosystems, CA, USA), as per manufacturer’s instructions, to test for false negatives due to lack of DNA amplification. Furthermore, the samples were also tested in the duplex format (Table 3) with *T*. *urticae*-specific and 18S internal control real-time PCR.

**Table 3:** Real-time PCR assay: Reaction composition and cycling conditions.

Reaction composition					
Component	Final concentration		Step	Temperature	
	Simplex	Duplex			
PerfecTA probe Mastermix	1 ×	1 ×	Initial denature	95°C	2 min
Primer F (Turit_1F)	300 nM	300 nM		35 cycles of	
Primer R (Turt_1R)	300 nM	300 nM	Denature	95°C	15 sec
Probe (Turti_1P, FAM)	250 nM	250 nM	Anneal/extension	62°C	45 sec
18S Forward	-	50 nM			
18S Reward	-	50 nM			
18S Probe (VIC)	-	50 nM			
BSA	0.25 μg/μL	0.25 μg/μL			
DNA template	1–20 ng	1–20 ng			
PCR grade water	adjust volume to 20 μL	adjust volume to 20 μL			

Note: Fluorescence signal was read in the FAM channel for the *T*. *urticae* PCR and VIC for 18S internal positive control at the end of each cycle during the cycling phase of the real-time PCR assay.

## Results

### 2.1 Real-time PCR assay design

Real-time PCR targeting *T*. *urticae* was developed from a suitable region of ITS1 gene. From an alignment of 109 ITS sequences (S2 Table), including 36 from *T*. *urticae* and 73 from closely related *Tetranychus* species, a region suitable for primers/probe design was identified. In the real-time PCR design, six morphologically confirmed *Tetranychus* species were used for DNA sequencing, including *T*. *kanzawai*, *T*. *ludeni*, *T*. *parakanzawai*, *T*. *pueraricola*, *T*. *truncatus* and *T*. *urticae*. The DNA sequences of *Tetranychus* species obtained in this study and from GenBank [7,17,24,31,37–40] were compared; unreliable sequences were excluded in the assay design. Total 17 *Tetranychus* species were used for sequence alignment and *in silico* testing in the design: *T*. *collyerae*, *T*. *evansi*, *T*. *ezoensis*, *T*. *kanzawai*, *T*. *lambi*, *T*. *ludeni*, *T*. *merganser*, *T*. *misumaiensis*, *T*. *neocaleodonicus*, *T*. *pacificus*, *T*. *parakanzawai*, *T*. *phaselus*, *T*. *piercei*, *T*. *pueraricola*, *T*. *truncatus*, *T*. *turkestani*, and *T*. *urticae* (S2 Table). A TaqMan probe based assay, specific to the sequence of *T*. *urticae* produced a 142 bp long amplicon. The nucleotide position for the primers and probe shown in brackets refer to the area of the ITS sequence of T. urticae voucher 128_2 Canary (NCBI Accession No. HM565888). Turti_1F: 5′-GTTTTACACTTCTTCGCCTAA-3′ (forward primer, 409–429); Turti-1R: 5′-CACCGCTTGAAGATGTATCT-3′ (reverse primer, 549–530) and Turti_1P: FAM-5′-CAATTGTTTTCAAACCCTCTCAATGC-3′-BHQ1 (probe, 467–492). Forward primers and probe sequence were tested against the ITS sequence alignment in Geneious, yielding no complete matches to non- *T*. *urticae* species.

### 2.2 Optimisation of PCR conditions

The real-time PCR assay was observed to be robust and reliable as six *T*. *urticae* DNA extracts were all successfully amplified with different commercial mastermixes tested (not shown). Similar *Cq* values were obtained for each sample among the commercial mastermixes used and there was no significant difference among the tests although slight intensity increases were observed in the mastermixes with additional 0.25 mM MgCl_2_ added. PerfeCTa qPCR Tough Mix (Quanta Bioscience) was chosen in the assay to further optimise the PCR conditions. The real-time assay was demonstrated to perform consistently well under different PCR conditions. There was negligible difference in the *Cq* values when the assay was run with different concentrations of primers (300 and 500 nM) or probe (150, 200 and 250 nM). The *Cq* values were not significantly different when the assay was tested at annealing/extension temperatures of 58, 60, 62 and 64°C. The optimal annealing/extension temperature was chosen as 62°C, the optimal primer and probe concentrations of 300 nM and 250 nM were selected, respectively (Table 3). Under the optimised condition, the assay tested in duplex format with 18S internal control (Table 3) did not show any obvious *Cq* values alteration.

### 2.3 Analytical and diagnostic specificity

All the *T*. *urticae* samples listed in Table 1 were successfully amplified by the real-time PCR assay, no amplifications were observed in the non-target *Tetranychus* species (Table 2). This assay had an analytical specificity of 100% for identifying *T*. *urticae* accurately and diagnostic specificity of 100% as no non-target closely related species amplified. The assay was able to accurately identify 20 *T*. *urticae* populations, from different countries: Australia, Colombia, India, Japan, Malaysia, Netherlands, New Zealand and USA on various hosts, *Malus domestica*, *Citrus unshiu*, *Erythrina* sp, *Pyrus* sp., *Phaseolus* sp. *Rosa* spp. and *Fragaria × ananassa* (Table 1). No template controls for the assay were all negative.

### 2.4 Analytical sensitivity and specificity

**Fig 1:**
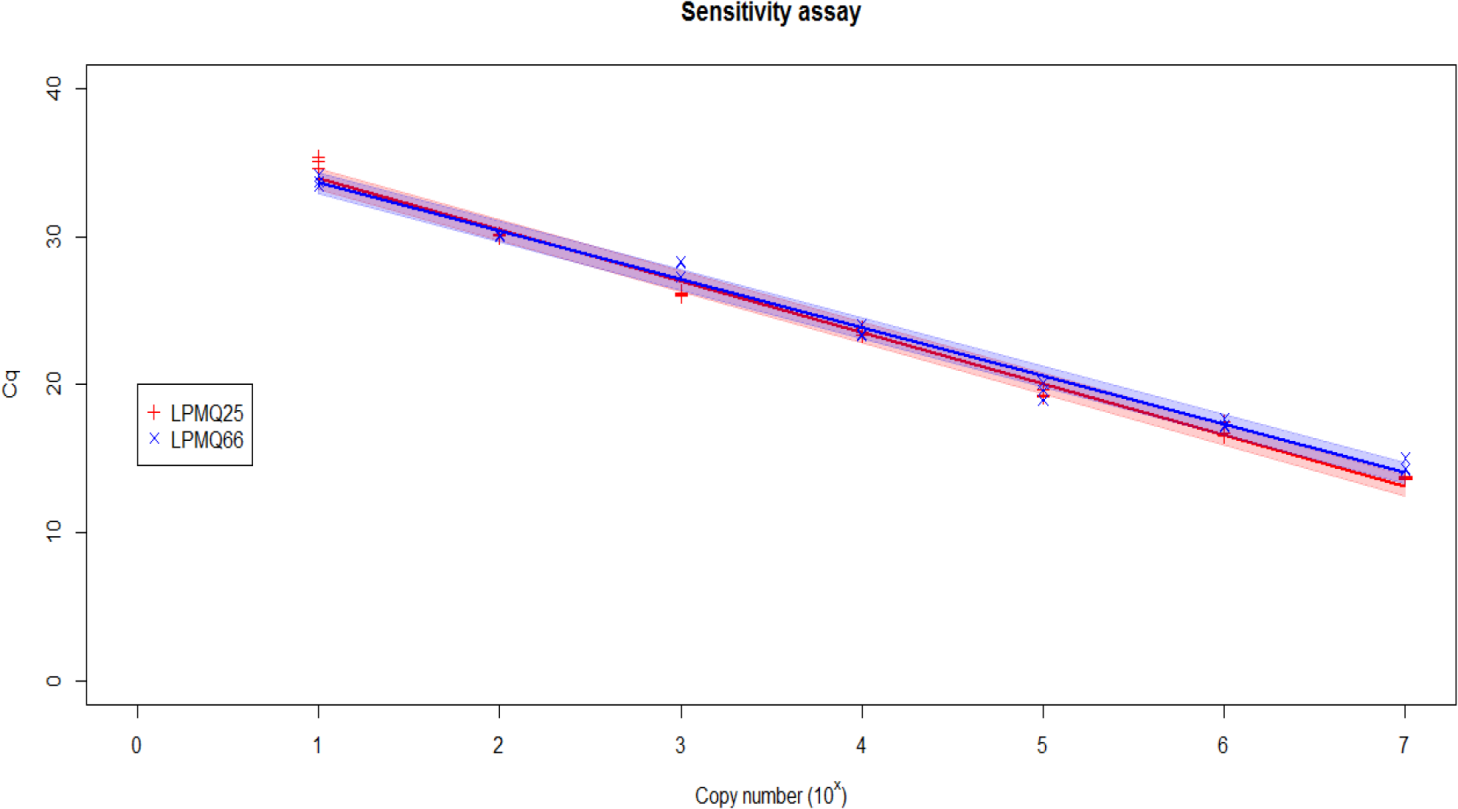
Sensitivity test of the real-time PCR assay for the identification of *T*. *urticae*. Plasmid containing ITS1 insert of *T*. *urticae* were series diluted to create calibration curves for sensitivity calculations. The standard curve built from *Cq* values against the log copy number (range = 10^7^–10 copies) of ITS1 insert (n = 3). The 95% confidence intervals of the slopes were plotted with a blue line for LPMQ66 and a red line for LPMQ25. The *r* ^*2*^ = 0.993 for LPMQ66 and 0.990 for LPMQ25 were obtained for the assay.

The linear dynamic range for the assay was tested on two plasmid DNAs containing the ITS1 inserts and extended from 10^7^–10 copies of plasmid DNA. The 95% confidence limits of the linear dynamic range were plotted in Fig 1. Amplification efficiencies for both plasmids were 98% with a strong correlation coefficient (r^2^ = 0.993 and 0.990%) (Fig 1).

The limit of detection (LOD) for the assay was estimated to be 10 copies of target DNA. A template concentration of 1 copy/reaction was sporadically detected in the assay with an average *Cq* value >35 cycles. The calibration curves shown in Fig 1 was able to detect 100% of the samples (and replicates) at the 10 copies/reaction. Sensitivity testing using a series dilution of DNA extracted from one mite showed that the assay could detect a 1 to 1000 dilution of the DNA extract (not shown).

The assay was determined to have high repeatability and reproducibility, with very low intra-run %CV from 0.16 to 1.33 (Table 3). Similarly low inter-run %CV of 0.36–2.02 (Table 4) were produced. The very low %CV (<5%) indicates the assay has high reproducibility and repeatability.

**Table 4:** Repeatability and reproducibility of the real-time PCR assay.

Sample ID	DNA Ext. methods^a^	Repeatability^b^ (%CV)		Reproducibility ^c^ (%CV)
		Run 1	Run 2	Run 1 ~ Run 2
Q10	QG	1.18	0.16	1.10
MZ5	ZR	0.29	0.74	0.96
Q25	QG	0.42	1.61	1.36
MQ54	QG	1.33	0.30	2.02
MK5	KF	0.30	0.52	0.36

Note:

^**a**^ all the DNA samples were extracted with one individual mite with different extraction methods, QG = Qiagen DNeasy Blood and Tissue kit; ZR = ZR Tissue & Insect DNA kit (Zymo Research, CA, USA); KF = Kingfisher Cell and Tissue kit (Thermo Scientific, USA).

^**b**^ Repeatability is calculated as the percent coefficient of variance (%CV) of *Cq*s of a sample within a single run.

^**c**^ Reproducibility is calculated as the percent coefficient of variance of *Cq*s of a sample across independent runs.

### 2.5 Blind panel testing

A total of twenty-five samples of mite species were provided in a blind panel assay. The tests were conducted with simplex and duplex formats, and similar *Cq* values were observed. The two independent tests produced consistent results, of those, 15/25 were identified accurately as *T*. *urticae* (Table 5). The identification of the rest of the samples as non-*T*. *urticae* was accurate. The test results were independently matched to the original identities of the samples provided.

**Table 5:** Blind panel validation of the real-time PCR assay.

	Real-time assay		Organism information			
Sample ID	Cq values	PCR results	Identity	Origin	Life stage	Host
M1	20.95	+	*T*. urticae*	USA	Adult	*Malus domestica*
M2	28.90	+	*T*. urticae*	Malaysia	Egg	*Gypsophila paniculata*
M3	N/A	-	*T*. neocaldonicus*	Fiji	Adult	*Moringa oleifera*
M4	25.07	+	*T*. urticae*	India	Nymph	*Rosa* spp.
M5	N/A	-	*Eotetranychus sexmaculatus*	New Zealand	Adult	*Aristolella serata (Makomako)*
M6	N/A	-	*Eotetranychus sexmaculatus*	New Zealand	Adult	*Aristolella serata (Makomako)*
M7	26.00	*+*	*T*. urticae*	India	Nymph	*Rosa* spp.
M8	N/A	-	*T*. ludeni*	India	Adult	*Rosa* spp.
M9	20.70	+	*T*. urticae*	India	Egg	*Rosa* spp.
M10	23.59	+	*T*. urticae*	India	Egg	*Rosa* spp.
M11	18.26	+	*T*. *urticae*	Australia	Adult	*Phasolus* sp.
M12	N/A	-	*T*. *ludeni*	Australia	Adult	*Dahlia* sp.
M13	N/A	-	*T*. *ludeni*	Australia	Adult	*Prunus persica*
M14	26.63	+	*T*. *urticae*	Colombia	Nymph	*Rosa* spp.
M15	N/A	-	*T*. *collyerae*	New Zealand	Adult	*Coprosma* sp.
M16	N/A	-	*T*. *collyerae*	New Zealand	Adult	*Coprosma* sp.
M17	20.05	+	*T*. *urticae*	India	Adult	*Rosa* spp.
M18	17.89	+	*T*. urticae*	Thailand	Adult	*Rosa* spp.
M19	22.32	+	*T*. *urticae*	China	Adult	* Salix matsudana f*. *tortuosa*
M20	19.09	+	*T*. *urticae*	China	Adult	* Salix matsudana f*. *tortuosa*
M21	19.47	+	*T*. *urticae*	China	Adult	* *Unknown
M22	21.32	+	*T*. *urticae*	China	Adult	Unknown
M23	N/A	-	*T*. *kanzawai*	China	Adult	* Gossypium* spp.
M24	19.83	+	*T*. urticae*	India	Adult	*Chrysanthemum* spp.
M25	N/A	-	*Paralamellobates* sp.	Philippines	Adult	*Musa* spp.

Samples denoted with asterisk (*) were identified using molecular methods. All other samples were identified morphologically and confirmed with DNA sequences.

## Discussion

*T*. *urticae* is a common interception and non-regulated pest at New Zealand borders, however, females are usually found in the sample, thus the species level identification could not be achieved. As a result of the genus level identification, the interception is categorised as a regulated organism by the New Zealand’s Biosecurity Organisms Register for Imported Commodities. As a consequence, shipments require treatment, normally methyl bromide fumigation. Therefore, the accurate and rapid identification of species is important for New Zealand exports and imports. A novel TaqMan real-time assay targeted on *T*. *urticae* was developed and it meets the requirement for border identification of *Tetranychus* interception. The resulting assay for *T*. *urticae* is highly specific, and unlikely to give false positives for other unrelated species. The routine application of this assay at New Zealand borders will assist the exporters/importers and their trading partners on quarantine decision making and treatment of their goods. The successful application of the assay will avoid unnecessary fumigation of the fresh produce, which will provide better quality of produce with longer shelf life and reduce the environmental and human health concerns associated with fumigation. The industry will benefit from reduced costs to import/export.

### 3.1 DNA sequences of *Tetranychus* species and assay design

With the increasing number of sequences being deposited in public databases, concerns about taxonomic misidentification of insect/invertebrate specimens is growing [41,42]. Misidentification of DNA sequences of *Tetranychus* species deposited in GenBank have been reported [7,43] from the recent wide studies on the genetic diversity and molecular identification of the species [3,4,7,17,18,23,24,31,44,45]. For example, Hinomoto et al. (2007) reported that 13 COI sequences deposited in GenBank as *T*. *urticae* were more likely *T*. *truncatus* [43], and de Mendonca et al. (2011) also showed that over 25% of ITS2 (26 out of 105) and 26% (36 out of 137) of COI from *Tetranychus* were misidentified [7]. When ITS sequences were aligned for the real-time PCR design, misidentified DNA sequences were also noticed. The sequence under accession number GQ141937 submitted in GenBank as *Tetranychus kanzawai* voucher NJAU-Acari-Te-ka0405FJ-01 was one of the examples; it aligned closely to *T*. *ludeni* and separated from *T*. *kanzawai*. The reasons for the misidentification are likely due to the limitation of the morphological features of the specimens and close relatedness of those species. The unreliability of DNA sequences in public databases restricts their direct use for molecular diagnostics [7]. Therefore it is crucial to manually verify DNA sequences downloaded from GenBank and to remove suspected misidentification sequences during assay design.

**Fig 2:**
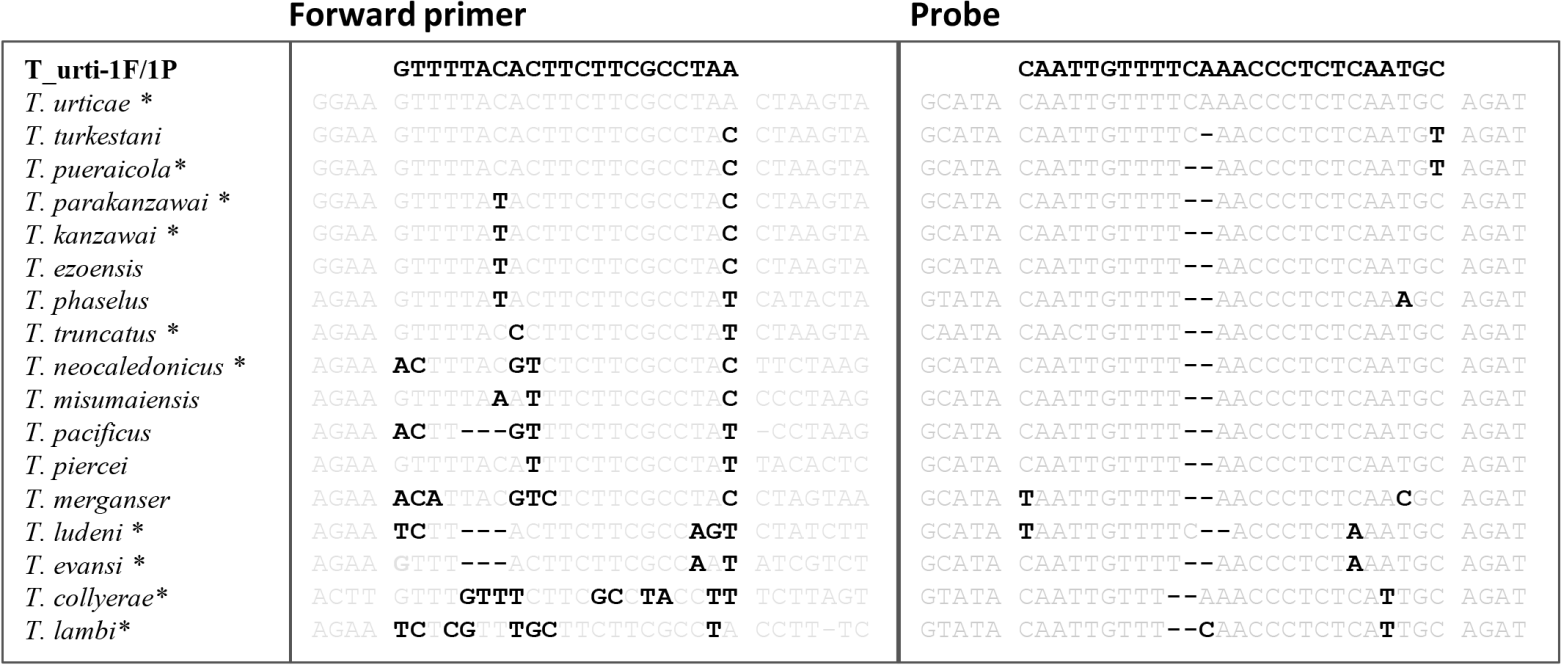
Alignment of the forward primer and probe regions of ITS1 sequences from *Tetranychus* species. The bold black letters indicated the SNPs and the dashed line indicated the indels. Asterisk (*) denotes the tested species in the real-time PCR assay. The reverse primer is not listed as there are no SNPs among the *Tetranychus* species compared.

Intra-species diversity of ITS sequences among *T*. *urticae* populations is low and their ITS sequences are completely homogenous worldwide [4,22,24]. Although ITS2 is a widely used marker for *Tetranychus* phylogeny and species discrimination [4,16,18,19], no suitable region could be chosen for *T*. *urticae*-specific primers and probe design. In comparison to ITS2, the ITS1 region is variable among *Tetranychus* species, but the intra-specific level diversity for *T*. *urticae* is low (<2%), thus it could enable us to identify a conserved region for *T*. *urticae* ITS1 sequence and provided the possibility to design a real-time PCR assay for targeting *T*. *urticae*. DNA sequence comparison showed that there are SNPs and indels in ITS1 sequences which could be targeted for primer and probe design. The ITS1 region could distinguish *T*. *urticae* from the highly similar species, *T*. *turkestani*, *T*. *kanzawai*, *T*. *parakanzawai*, *T*. *pueriricola* and *T*. *truncatus*. As a result, the *T*. *urticae*-specific real-time PCR assay was designed by targeting the ITS1 region, with SNPs and indels present in non-target *Tetranychus* spp. sequences in the forward primer and probe (Fig 2). This real-time PCR assay successfully detected the *T*. *urticae* species and did not cross-react with other closely related species tested. It could detect *T*. *urticae* from various countries (Tables 1 and 4), thus indicating this real-time PCR assay could be able to apply to research and biosecurity agencies worldwide in detection of *T*. *urticae*.

In addition, analysis of COI sequences of *Tetranychus* species showed; 1) high AT content, which was also previously observed in the other tetranychid mites [6,17,46]; 2) high intra-specific variation among *T*. *urticae* species; 3) high sequence identity between *T*. *turkestani* and *T*. *urticae* [7,8]. Therefore, COI is not an ideal target for designing a *T*. *urticae*-specific real-time PCR assay. Moreover, D2/D3 region was also analysed and no suitable region could be identified because of the high sequence identity among different *Tetranychus* species.

### 3.2 Performance, validation and application of the real-time PCR assay for *T*. *urticae* detection

Application of the real-time assay in the New Zealand quarantine framework provides an alternative to morphological identification, especially when only female or immature stages of the mites are available. The real-time PCR assay targeting *T*. *urticae* developed in this study is conformant with the MIQE guidelines for qualitative assays [47]. MIQE guidelines provide assay performance criteria such as amplification efficiency, linear dynamic range, limit of detection, and analytical and diagnostic specificity. High specificity is much desired in border diagnostics and was fulfilled by this assay. The assay showed high efficiency and sensitivity in detecting the target species. We tested a total of 63 samples (Tables 1 and 2) for specificity and an additional 25 samples (Table 5) during the blind panel validation, including the closely related species, *T*. *neocaledonicus*, *T*. *kanzawai*, *T*. *parakanzawai*, *T*. *pueraricola*, *T*. *truncatus*, *T*. *ludeni*, and *T*. *evansi*. No cross reaction with those species was observed (Tables 2 and 5). For example, no cross-reaction was observed in the most closely related species, *T*. *pueraricola* (Table 2), in which there is 1 SNP in the forward primer and an indel in the probe, either in *T*. *kanzawai* and *T*. *parakanzawai* species where there are 2 SNPs in the forward primer and 1 indel in the probe (Fig 2). *T*. *turkestani* and *T*. *urticae* are closely related species; however, several attempts to obtain *T*. *turkestani* specimens were not successful, thus it was not included in the tests. In spite of being unable to test *T*. *turkestani* specimens, *in silico* analysis of the *T*. *urticae*-specific real-time assay showed that there was a mismatch in the crucial position at the 3′-end of the forward primer, and 1 indel and 1 mismatch in the 3′-end region of the probe in *T*. *turkestani* ITS sequences (Fig 2). Combining the mismatches in the primer and probe sequences and the stringent real-time PCR conditions, it is unlikely that the real-time PCR assay for *T*. *urticae* will cross-react with *T*. *turkestani*. Indeed, the real-time PCR assay was designed based on the current available ITS sequences from the databases and in-house, and validated with the *Tetranychus* species we could obtain, therefore further tests will be conducted if more *Tetranychus* species intercepted and DNA sequences available online.

PCR-RFLP protocols have been used to identify *Tetranychus* species [24,25], however, this method is time consuming, involving more handling steps. In contrast, the real-time PCR assay combines the annealing and extension steps of the reaction, making it a significantly faster approach than conventional PCR or PCR-RFLP, typically taking less than 90 minutes to perform a 40-cycle reaction. Furthermore, the real-time assay is a closed-tube system which does not require any post-PCR manipulations such as agarose gel electrophoresis; this helps to reduce the risk of cross contamination between samples. Therefore, this assay would enable quick, reliable, and robust testing of large numbers of samples, and can be implemented in a high-throughput platform with minimal modification. The newly developed assay has shown to be repeatable and reproducible and worked well with different DNA extraction methods. Differences in the *Cq* values obtained from the tested samples were more likely the result of differences in the quality of the tissue material used for DNA extraction and the amounts of DNA obtained, rather than effectiveness of the tests (Table 1).

Overall, the assay can be directly applied in a quarantine framework. It has great advantages over morphological keys; although a single PCR assay may take longer to perform than a morphological identification, the technique scales easily to identify dozens, or potentially hundreds of specimens in a single operation. PCR assays are also easily transferable, a scientist or diagnostician capable of performing PCR can perform any real-time PCR assay without specific training. Moreover, the assay is faster than PCR-RFLP and meets New Zealand border entomological identification requirements.

## Conclusion

Real-time PCR based assays may provide a much needed alternative to barcoding and other PCR-based molecular diagnostic techniques used for border security. This developed real-time assay has been used successfully to detect *T*. *urticae* from different geographical regions and hosts, and therefore provides highly sensitive, rapid and accurate detection of interceptions with various biosecurity applications. The assay was also demonstrated to be highly specific and reliable, regardless of the reagent kits used. It is suitable for routine diagnostics, facilitating exports and imports, as well as in aiding border security agencies worldwide. The applications of this assay will facilitate quarantine decision making and benefit trading partners.

## Supporting Information

S1 Table Primer used for PCR and sequencing of the COI and ITS gene regions. (DOCX)

S2 Table ITS sequences used in the alignment for the real-time PCR assay design, which included those downloaded from GenBank and sequenced by this study. (DOCX)
