# Supplementary material for: Development and Validation of a Real-Time PCR Assay for Rapid Detection of Two-Spotted Spider Mite, Tetranychus urticae (Acari: Tetranychidae)
Source: PLoS One. 2015 Jul 6;10(7):e0131887. doi: 10.1371/journal.pone.0131887 (PMC4492583; doi:10.1371/journal.pone.0131887)
Supplement: S1 Table — (DOCX) [file pone.0131887.s001.docx]

**S1 Table.** Primer used for PCR and sequencing of the COI and ITS gene regions

| Name | Gene | Sequence (5’ to 3’) | References | Comment |
| --- | --- | --- | --- | --- |
| CI J 1718F | COI | GGAGGATTTGGAAATTGATTAGTTCC | [[30](#_ENREF_30)] | PCR and sequencing |
| COIVERA | COI | GATAAAACGTAATGAAAATGAGCTAC | [[31](#_ENREF_31)] | PCR and sequencing |
| 18SF1 | ITS | 5-AGAGGAAGTAAAAGTCGTAACAAG-3 | [[27](#_ENREF_27)] | PCR and sequencing |
| HC2R | ITS | 5-ATATGCTTAAATTCAGCGGG-3 | [[4](#_ENREF_4)] | PCR and sequencing |
| InternS | ITS | 5-GATCACTCGAATTACCAATCG-3 | [[28](#_ENREF_28)] | for seq. |
| InternSRev | ITS | CGATTGGTAATTCGAGTGATC | [[28](#_ENREF_28)] | for seq. |
| Mite B | ITS1 | GCTGCGTTCTTCATCGATC | [[29](#_ENREF_29)] | PCR and sequencing |
| Prime C | ITS1 | GAGGAAGTAAAAGTCGTAACAAGG | [[29](#_ENREF_29)] | PCR and sequencing |
