# Supplementary material for: Development and Validation of a Real-Time PCR Assay for Rapid Detection of Two-Spotted Spider Mite, Tetranychus urticae (Acari: Tetranychidae)
Source: PLoS One. 2015 Jul 6;10(7):e0131887. doi: 10.1371/journal.pone.0131887 (PMC4492583; doi:10.1371/journal.pone.0131887)
Supplement: S2 Table — (DOCX) [file pone.0131887.s002.docx]

**S2 Table**. ITS sequences used in the alignment for the real-time PCR assay design

| **Species** | **ACC#** | **Length** | **Origin** | **Reference** |
| --- | --- | --- | --- | --- |
| *T. evansi* | AB735996 | 824 | Japan: Tokyo | [[17](#_ENREF_17)] |
| *T. evansi* | AB735997 | 823 | Japan: Kagoshima | [[17](#_ENREF_17)] |
| *T. evansi* | AB738755 | 1162 | Japan: Chiba, Narita | [[24](#_ENREF_24)] |
| *T. evansi* | FJ440673 | 1191 | Brazil: Piracicaba | [[31](#_ENREF_31)] |
| *T. evansi* | GU145105 | 1191 | Argentina | [[37](#_ENREF_37)] |
| *T. evansi* | FJ440674 | 1191 | Taiwan: Wufeng | [[31](#_ENREF_31)] |
| *T. takafujii* | AB257746 | 1162 | Japan: Kyoto | Osakabe, Kotsubo et al. 2006. unpub. |
| *T. evansi* | AB257747 | 1162 | Japan: Kyoto | Osakabe, Kotsubo et al.2006. unpubl. |
| *T_evansi* | AM408033 | 1218 | Unknown | [[39](#_ENREF_39)] |
| *T_evansi* | KP744535 | 549 | Australia | This study |
| *T. ludeni* | KP744529 | 1165 | Japan | This study |
| *T. ludeni* | AB076371 | 1197 | Japan: Wakayama, Kishigaw | Osakabe, Kotsubo 2006. unpubl. |
| *T. ludeni* | AB736009 | 814 | Japan: Okinawa | [[17](#_ENREF_17)] |
| *T. ludeni* | AB736008 | 814 | Japan: Ibaraki | [[17](#_ENREF_17)] |
| *T. ludeni* | AB736010 | 814 | Japan: Chiba | [[17](#_ENREF_17)] |
| *T. ludeni* | AB738754 | 1154 | Colombia | [[24](#_ENREF_24)] |
| *T. lambi* | KP744533 | 514 | Unknown | This study |
| *T. phaselus* | AB736023 | 826 | Japan: Ibarak | [[17](#_ENREF_17)] |
| *T. phaselus* | AB736024 | 826 | Japan: Okinawa | [[17](#_ENREF_17)] |
| *T. phaselus* | AB257733 | 1171 | Japan: Ibaraki, Ushiku | Osakabe, Kotsubo et al. 2006. unpub. |
| *T. phaselus* | AB257734 | 1171 | Japan: Ibaraki, Ushiku | Osakabe, Kotsubo et al. 2006. unpub. |
| *T. phaselus* | AB257735 | 1171 | Japan: Ibaraki, Ushiku | Osakabe, Kotsubo et al. 2006. unpub. |
| *T. phaselus* | AB738751 | 1171 | Japan: Kanagawa, Yokohama | [[24](#_ENREF_24)] |
| *T. piercei* | AB736025 | 831 | Japan: Okinaw | [[17](#_ENREF_17)] |
| *T. piercei* | AB736026 | 831 | Japan: Okinaw | [[17](#_ENREF_17)] |
| *T. piercei* | AB736027 | 831 | Japan: Okinaw | [[17](#_ENREF_17)] |
| *T. piercei* | AB257748 | 1178 | Japan: Ibaraki, Tsukuba | Osakabe, Kotsubo et al. 2006. unpub |
| *T. piercei* | AB738750 | 1178 | Malaysia | [[24](#_ENREF_24)] |
| *T. mergans* | AB738753 | 1181 | Mexico | [[24](#_ENREF_24)] |
| *T. misumaiensis* | AB736011 | 822 | Japan: Hokkaido | [[17](#_ENREF_17)] |
| *T. neocaledonicus* | AB736013 | 809 | Japan: Okinawa | [[17](#_ENREF_17)] |
| *T. neocaledonicus* | AB736014 | 809 | Japan: Okinawa | [[17](#_ENREF_17)] |
| *T. neocaledonicus* | AB736012 | 809 | Japan: Tokyo | [[17](#_ENREF_17)] |
| *T. neocaledonicus* | AB738752 | 1163 | Japan: Tokyo | [[24](#_ENREF_24)] |
| *T. neocaledonicus* | KP744534 | 490 | Fiji | This study |
| *T. collyerae* | KP744532 | 578 | New Zealand | This study |
| *T. pacificus* | AB738744 | 1160 | USA | [[24](#_ENREF_24)] |
| *T. ezoensis* | AB735999 | 818 | Japan: Ibaraki | [[17](#_ENREF_17)] |
| *T. ezoensis* | AB257728 | 1161 | Japan: Iwate, Morioka | Osakabe, Kotsubo et al. 2006. unpub. |
| *T. ezoensis* | AB735998 | 818 | Japan: Iwate | [[17](#_ENREF_17)] |
| *T. kanzawai* | AB076370 | 1230 | Japan: Shizuoka, Kanaya | [[40](#_ENREF_40)] |
| *T. kanzawai* | AB736007 | 818 | Japan: Tokyo | [[17](#_ENREF_17)] |
| *T. kanzawai* | AB736003 | 818 | Japan: Okinawa | [[17](#_ENREF_17)] |
| *T. kanzawai* | AB736000 | 818 | Japan: Shizuoka | [[17](#_ENREF_17)] |
| *T. kanzawai* | AB736006 | 818 | Japan: Hokkaido | [[17](#_ENREF_17)] |
| *T. kanzawai* | AB736001 | 818 | Japan: Ibaraki | [[17](#_ENREF_17)] |
| *T. kanzawai* | AB736002 | 818 | Japan: Hokkaido | [[17](#_ENREF_17)] |
| *T. kanzawai* | AB736004 | 818 | Japan: Hokkaido | [[17](#_ENREF_17)] |
| *T. kanzawai* | AB736005 | 818 | Japan: Hokkaido | [[17](#_ENREF_17)] |
| *T. kanzawai* | AB738748 | 1161 | Taiwan | [[24](#_ENREF_24)] |
| *T. kanzawai* | KP744527 | 1172 | Japan | This study |
| *T. parakanzawai* | AB736020 | 818 | Japan: Okinawa | [[17](#_ENREF_17)] |
| *T. parakanzawai* | AB736018 | 818 | Japan: Okinawa | [[17](#_ENREF_17)] |
| *T. parakanzawai* | AB736017 | 818 | Japan: Ibaraki | [[17](#_ENREF_17)] |
| *T. parakanzawai* | AB736019 | 818 | Japan: Hokkaido | [[17](#_ENREF_17)] |
| *T. parakanzawai* | AB736022 | 818 | Japan: Chiba | [[17](#_ENREF_17)] |
| *T. parakanzawai* | AB736021 | 818 | Japan: Okinawa | [[17](#_ENREF_17)] |
| *T. parakanzawai* | AB257745 | 1161 | Japan: Ibaraki, Ami | Osakabe, Kotsubo et al. 2006. unpub. |
| *T. parakanzawai* | KP744524 | 1172 | Japan | This study |
| *T. pueraicola* | AB076372 | 1208 | Japan: Ibaraki, Hitachiohta | [[40](#_ENREF_40)] |
| *T. pueraicola* | AB736028 | 819 | Japan: Ibaraki | [[17](#_ENREF_17)] |
| *T. pueraicola* | AB736030 | 819 | Japan: Nara | [[17](#_ENREF_17)] |
| *T. pueraicola* | AB736029 | 819 | Japan: Nagano | [[17](#_ENREF_17)] |
| *T. pueraicola* | KP744528 | 1175 | Japan | This study |
| *T. truncatus* | JN018058 | 1033 | Bangladesh | Jahan, Tin, et al. 2011. unpub. |
| *T. truncatus* | JN018057 | 1197 | Bangladesh | Jahan, Tin, et al. 2011. unpub. |
| *T. truncatus* | AB736032 | 819 | Japan: Kyoto | [[17](#_ENREF_17)] |
| *T. truncatus* | AB736031 | 819 | Japan: Kyoto | [[17](#_ENREF_17)] |
| *T. truncatus* | AB257729 | 1163 | Japan: Kyoto | Osakabe, Kotsubo et al. 2006. unpub. |
| *T. truncatus* | AB257730 | 1164 | Japan: Kyoto | Osakabe, Kotsubo et al. 2006. unpub. |
| *T. truncatus* | KP744526 | 1174 | Japan | This study |
| *T. turkestani* | AM408032 | 1223 | Unknown | [[39](#_ENREF_39)] |
| *T. turkestani* | AB738745 | 1166 | USA | [[24](#_ENREF_24)] |
| *T. urticae* | AM408031 | 1220 | Spain? | [[39](#_ENREF_39)] |
| *T. urticae* | AM408035 | 1163 | Unknown | [[39](#_ENREF_39)] |
| *T. urticae* | AB076369 | 1205 | Japan: Wakayama, Gobo | [[40](#_ENREF_40)] |
| *T. urticae* | AB736033 | 820 | Japan: Hokkaido | [[17](#_ENREF_17)] |
| *T. urticae* | AB736034 | 821 | Japan: Ibaraki | [[17](#_ENREF_17)] |
| *T. urticae* | AB736035 | 819 | Japan: Okinawa | [[17](#_ENREF_17)] |
| *T. urticae* | AB736036 | 821 | Japan: Nagano | [[17](#_ENREF_17)] |
| *T. urticae* | AB736037 | 821 | Japan: Kanagawa | [[17](#_ENREF_17)] |
| *T. urticae* | AB736038 | 821 | Japan: Niigata | [[17](#_ENREF_17)] |
| *T. urticae* | AB738746 | 1164 | Uganda | [[24](#_ENREF_24)] |
| *T. urticae* | AB738747 | 1164 | Spain | [[24](#_ENREF_24)] |
| *T. urticae* | AM408030 | 1123 | Castellon | [[39](#_ENREF_39)] |
| *T. urticae* | KF544954 | 1163 | China: Nanjing | [[38](#_ENREF_38)] |
| *T. urticae* | KF544955 | 1163 | China: Shihezi | [[38](#_ENREF_38)] |
| *T. urticae* | HM565885 | 1209 | Spain: ES, Canary, Granadilla de Abona | [[7](#_ENREF_7)] |
| *T. urticae* | HM565878 | 1209 | France: FR, La Gaude-Bord du Var | [[7](#_ENREF_7)] |
| *T. urticae* | HM565886 | 1207 | Spain: ES, Canary, Arico | [[7](#_ENREF_7)] |
| *T. urticae* | HM565887 | 1199 | Spain: ES, Canary, Arico | [[7](#_ENREF_7)] |
| *T. urticae* | HM565879 | 1207 | France: FR, Nice | [[7](#_ENREF_7)] |
| *T. urticae* | HM565888 | 1200 | Spain: ES, Canary, Aguimes | [[7](#_ENREF_7)] |
| *T. urticae* | HM565875 | 1160 | France: FR, Perpignan | [[7](#_ENREF_7)] |
| *T. urticae* | HM565889 | 1205 | Greece: GR, Ieraptera | [[7](#_ENREF_7)] |
| *T. urticae* | HM565877 | 1204 | France: FR, Saint Nazaire | [[7](#_ENREF_7)] |
| *T. urticae* | HM565880 | 1147 | Spain: ES, Vilademuls/Galliners | [[7](#_ENREF_7)] |
| *T. urticae* | HM565881 | 1191 | Spain: ES, Bordils/Juia | [[7](#_ENREF_7)] |
| *T. urticae* | HM565883 | 1198 | Spain: ES, S. Andreu de Llavaneres | [[7](#_ENREF_7)] |
| *T. urticae* | HM565874 | 1205 | France: FR, Elne | [[7](#_ENREF_7)] |
| *T. urticae* | HM565876 | 1152 | Spain: SP, S.Susanna/Cami de la Riera | [[7](#_ENREF_7)] |
| *T. urticae* | HM565884 | 1198 | Spain: ES, Canary, Santiago del Teide | [[7](#_ENREF_7)] |
| *T. urticae* | HM565882 | 1185 | Spain: ES, Bordils/Juia | [[7](#_ENREF_7)] |
| *T. urticae* | HM565873 | 1210 | France: FR, Elne | [[7](#_ENREF_7)] |
| *T. urticae* | KP744522 | 1173 | Japan | This study |
| *T. urticae* | KP744523 | 1142 | Japan | This study |
| *T. urticae* | KP744525 | 1173 | Japan | This study |
| *T. urticae* | KP744530 | 1175 | Japan | This study |
| *T. urticae* | KP744531 | 1180 | Japan | This study |
